# Predictors of Athlete’s Performance in Ultra-Endurance Mountain Races

**DOI:** 10.3390/ijerph18030956

**Published:** 2021-01-22

**Authors:** Pedro Belinchón-deMiguel, Pablo Ruisoto, Beat Knechtle, Pantelis T. Nikolaidis, Beliña Herrera-Tapias, Vicente Javier Clemente-Suárez

**Affiliations:** 1 Department of Nursing and Nutrition, Faculty of Biomedical and Health Sciences, Universidad Europea de Madrid, 28670 Villaviciosa de Odón, Spain; pedro.belinchon@universidadeuropea.es; 2 Department of Health Sciences, Public University of Navarre, 31006 Pamplona, Spain; pablo.ruisoto@unavarra.es; 3 Institute of Primary Care, University of Zurich, 8001 Zurich, Switzerland; beat.knechtle@hispeed.ch; 4 Gesundheitszentrum St. Gallen, 9001 St. Gallen, Switzerland; 5 School of Health and Caring Sciences, University of West Attica, 12243 Egaleo, Greece; pademil@hotmail.com; 6 Exercise Physiology Laboratory, 18450 Nikaia, Greece; 7 Grupo de Investigación en Derecho, Política y Sociedad, Universidad de la Costa, 080002 Barranquilla, Colombia; bherrera3@cuc.edu.co; 8 Faculty of Sports Sciences, Universidad Europea de Madrid, 28670 Villaviciosa de Odón, Spain; 9 Grupo de Investigación en Cultura, Educación y Sociedad, Universidad de la Costa, 080002 Barranquilla, Colombia

**Keywords:** psychology, odontology, nutrition, training, stress, running

## Abstract

Background: In previous studies, ultra-endurance performance has been associated with training and psychological variables. However, performance under extreme conditions is understudied, mainly due to difficulties in making field measures. Aim: The aim of this study was to analyze the role of training, hydration, nutrition, oral health status, and stress-related psychological factors in athletes’ performance in ultra-endurance mountain events. Methods: We analyzed the variables of race time and training, hydration state, nutrition, oral health status, and stress-related psychological factors in 448 ultra-endurance mountain race finishers divided into three groups according to race length (less than 45 km, 45–90 km, and greater than 90 km), using a questionnaire. Results: Higher performance in ultra-endurance mountain races was associated with better oral health status and higher accumulative altitude covered per week as well as higher positive accumulative change of altitude per week during training. In longer distance races, experience, a larger volume of training, and better hydration/nutrition prior to the competition were associated with better performance. Conclusions: Ultra-endurance mountain athletes competing in longer races (>90 km) have more experience and follow harder training schedules compared with athletes competing in shorter distances. In longer races, a larger fluid intake before the competition was the single best predictor of performance. For races between 45 and 90 km, training intensity and volume were key predictors of performance, and for races below 45 km, oral health status was a key predictor of performance. Psychological factors previously reported as ultra-endurance mountain race performance predictors were inconsistent or failed to predict the performance of athletes in the present research.

## 1. Introduction

Participation in ultra-endurance mountain events has shown an exponential increase in recent years [1]. Specific research in this extreme endurance event has shown an increase in protein catabolism and muscle degradation [2,3], an increased erythropoiesis to compensate exercise-induced hemolysis [4], an accumulation of blood lactate below the anaerobic threshold [5], and an increase in the consumption of fat [6].

Ultra-endurance mountain events are highly stressful, usually performed at 71% of maximum heart rate (HR) [7] and characterized by an intense sympathetic modulation evaluated on the basis of heart rate variability (HRV) [8]. Regarding the training programs, the literature has highlighted the importance of training schedule (speed and volume), the speed in half-marathon and marathon to attain ‘top’ performance in ultra-endurance races [9].

A higher performance in ultra-endurance events has been associated with two physiological factors, i.e., low body fat index [10,11] and an appropriate hydration and nutritional status [8,11,12]. In this respect, it was found that successful ultra-endurance mountain athletes drank enough to maintain an optimal performance during the race [12]. In addition, an appropriate fuel support is essential for performance, since an extreme negative energy balance was reported in previous ultra-endurance mountain events [8]. However, nutritional and fluid intake during ultra-endurance events presents a risk for oral health. In fact, the absence of oral health care and the presence of caries, dental erosion, and periodontal disease [13,14,15,16] are directly related to the extensive use of sports drinks, gels, and energy bars [17,18]. In this line, the negative effect of acid and high-carbohydrate foods aggravated by a decreased saliva flow rate during intense exercise [19,20] can negatively affect health and performance in ultra-endurance mountain athletes.

Previous literature on athletes’ performance in these events has also focused on the analysis of ergogenic aids, showing the extensive consumption of nonsteroidal anti-inflammatory drugs (NSAIDs) or central nervous system activators such as caffeine [1,21]. In addition, different psychological factors such as higher pain tolerance [22,23] or better stress management skills [24,25] have also been associated with better performance.

The role of multifactorial parameters in ultra-endurance mountain performance is still poorly known or understood. For this reason, we conducted the present research with the aim to analyze the role of training schedule, hydration, nutrition, oral health status, and stress-related psychological factors in athletes’ performance in ultra-endurance mountain events. The initial hypothesis was that successful athletes would present a different training schedule, hydration, nutrition, oral health, and stress-related psychological profile compared with non-successful athletes.

## 2. Materials and Methods

### 2.1. Participants

A total of 448 ultra-endurance mountain race finishers were analyzed. According to the three types of ultra-endurance mountain races, based on race length, that athletes can perform, they were divided into three groups: G1, athletes involved in ultra-endurance mountain races below 45 km (*n* = 234; 37.97 ± 7.31 years; 1.7315 ± 0.1341 m; 71.40 ± 10.38 kg); G2, athletes involved in ultra-endurance mountain races between 45 and 90 km (*n* = 79; 39.26 ± 6.92 years; 1.7535 ± 0.0731 m; 73.86 ± 10.61 kg); and G3, athletes involved in ultra-endurance mountain races longer than 90 km (*n* = 135; 41.09 ± 7.26 years; 1.7557 ± 0.0713 m; 71.45 ± 8.01 kg). The three groups did not differ in age, body mass index, or total years of experience as ultra-endurance athletes. The average rate of successful (finisher) and non-successful (non-finisher) athletes in G1 was 74%, in G2 61%, and in G3 40%. Participants were recruited when they presented the documentation attesting their participation in the races. The inclusion criterion was that they were official participants in the races, and the exclusion criterion was that participants reported a medical condition or were taking medication. We obtained written consent from each participant in accordance with the Declaration of Helsinki, and the study was approved by the University Bioethics Committee (CIPI/002/17).

Athletes participated in the following races: Valls d’Aneu Ultratrail, 92 km-long and with 7344 m of accumulated positive altitude change, Emmona Ultratrail, 130 km-long and with 10,194 m of accumulated positive altitude change, Canfranc-Canfranc Ultra-endurance Mountain Race, 100 km-long and with 8848 m of accumulated positive altitude change, Great Trail of Peñalara, 114 km-long and with 5100 m of positive altitude change, Gruseg Trail, 51 km-long and with 2516 m of accumulated positive altitude change, and Extremadura Mountain Federation Circuit, including races from 6 km to 72 km and positive altitude changes from 910 m to 6133 m.

### 2.2. Design and Procedure

Data in the following self-reported variables were collected for all participants 24 h before the races at the race place. The questionnaire took about 10 min to complete. All participants in the races were invited to answer the questionnaire, and only those who accepted were registered. After the races, the race time was also recorded, as reported in the official classification of each race.

#### 2.2.1. Training, Hydration, and Nutrition Variables

We analyzed variables related to athletes’ experience in mountain races (years), total race time in the last marathon (min) and half-marathon (min), training schedule (volume, intensity, and accumulative altitude change), expected race time (min), difference between expected and real time (min), stretching time per week (min), and stretching sessions’ duration per week.

Regarding nutrition and hydration habits before the competitions, the following parameters were evaluated: intake of carbohydrate-based meals during the week of competition and fluid intake before the competition day (l). Finally, regarding the oral health status, the frequency of the following occurrences was recorded: grinding or clenching teeth while performing usual tasks, nibbling objects, biting nails, consuming vitamin C pills, having a wet pillow upon awakening, and burping repeatedly.

#### 2.2.2. Psychological Variables

We analyzed perceived stress, measured by the Perceived Stress Scale (PSS-14) [26], and general mental health status, measured by the Acceptance and Action Questionnaire, (AAQ-II) [27]. 

#### 2.2.3. Performance Race Variables

We analyzed the variables of total time in ultra-endurance mountain races (min), total distance (km), average running speed (km/h), accumulated positive and negative altitude (m), difficulty coefficient (total km × accumulated positive altitude change/1000), and the speed/difficulty coefficient ratio.

### 2.3. Statistical Analysis

Data were analyzed using the Statistical Package for the Social Sciences (SPSS) version 21 (SPSS Inc., Chicago, IL, USA). Kolmogorov–Smirnov tests were performed to test normality and homogeneity of each variable. A MANOVA was conducted to analyze differences between the three groups (G1, G2, G3) regarding the dependent variables previously described. Tukey tests were conducted for post hoc comparisons. In addition, the athletes in each group were divided in higher- and lower-performance subgroups by percentile 50 according to their race time, to analyze differences between performances in the variables analyzed. For this, an independent *t* test was used. The significance level was set at *p* < 0.05 in all analysis.

## 3. Results

No significant differences were found in any variable between G1 and G2 (Table 1). However, ultra-endurance mountain athletes in G3 recorded more experience, training sessions, hours per week, minutes per session, accumulated positive altitude per session and per week, compared with G1 and G2 athletes. No differences were found in psychological variables between the groups.

Table 2 shows differences in performance predictors for athletes competing in the three ultra-endurance mountain race distances. For G1, better performance was associated with higher speed training per week, but not with differences in psychological or nutritional/hydration status. For G2, higher performance was associated with higher training speed per week and more training sessions per week, but no differences were found for the other variables. For G3, higher performance was associated with higher intake of fluids before the competition, with no differences for the training and psychological variables.

## 4. Discussion

The aim of the present study was to analyze the role of training, hydration, nutrition, oral health status, and stress-related psychological factors in athletes’ performance in ultra-endurance mountain events. The initial hypothesis was partially confirmed, since differences in training, hydration, nutrition, and oral heath parameters were found for athletes depending on their performance and competition distance, but no differences appeared in stress-related psychological factors. 

Previous research found that half-marathon and marathon race times predicted race performance in ultra-endurance mountain races [9]. In the current research, we found that G1 athletes and G2 athletes with a better performance had significantly faster marathon times. Better performance in mountain races was associated with faster half-marathon times, which is in line with previous research [9,28]. Higher performance in ultra-endurance mountain races for distances below 90 km was directly linked to three training-related variables, i.e., training intensity and higher speed in shorter races, as previously reported in the literature [29,30]; training volume and density (sessions per week), since a large training volume may be necessary to obtain physiological adaptations in aerobic and anaerobic threshold intensities—which are the intensity zones involved in ultra-endurance race performance [31]—in line with previous research in ultra-endurance mountain events [1,29], though recent studies propose that ultra-endurance runners would benefit from incorporating high-intensity interval training in a low-volume training program [32,33,34,35]; higher positive change of altitude accumulated per week. Previous studies have suggested that the mechanical work performed on a positive slope may improve musculature, joint resistance, the resistance of ligamentous and tendinous tissues, and the effort tolerance of the locomotor system, which are critical for successful adaptation to the long-term efforts involved in ultra-endurance mountain events [36,37]. Therefore, it would be recommended to introduce more strength training sessions performed with a higher load in future training interventions for ultra-endurance mountain athletes [38].

Higher performance in ultra-endurance mountain races for distances over 90 km was linked to two main variables. Firstly, a higher fluid intake before the race, a result that highlights the importance of hydration in these races, consistent with previous studies that showed that the hydration status has a positive effect on thermoregulation and muscular function, factors crucial in extreme sport competitions [39]. It is important to note that a large fluid ingestion during ultra-endurance races could have a negative effect on performance, since inappropriate hydration can lead to hyper-hydration, and consequently to hyponatremia because of electrolyte imbalance, producing alterations in athletes’ cardiovascular response and performance, or to dehydration which also compromises the athletes’ health and performance. Secondly, the influence on performance of a larger experience in ultra-endurance mountain races is a factor that has been identified in earlier studies [1]. Interestingly, athletes with higher experience reported a more accurate self-perception of their performance, which indicates that athletes with a lower performance have a poorer knowledge of their own capacities [40].

Regarding the role of psychological variables in predicting athlete’s performance, no differences between groups or performance based on perceived stress or general mental health were observed. One possible explanation is that most participants reported very low psychological stress levels and good general mental health, on the basis of the evaluation scale established for the general population (M = 18.51, SD = 7.05) [41]. Therefore, the collected data may have failed to detect effects because of the lack of variability of our sample, which is not representative of the general population. In addition, in the case of ultra-endurance mountain races below 45 km, better oral health parameters were associated with higher performance. This result is in line with previous research conducted in elite athletes [14,16], highlighting the importance of an optimal oral health status for correct physiological functioning, especially in individuals participating in these extreme races.

A limitation of the present study is that we analyzed distances of <45, 45–90, and >90 km; thus, the findings should be generalized with caution to athletes competing in longer distances. Another limitation is the use of a self-reported questionnaire. On the other hand, a strength of this study is that it analyzed a relatively large sample and examined the role of many performance predictors such as training, hydration, nutrition, oral health, and stress-related psychological factors. These findings are expected to have large practical applications for practitioners working with ultra-endurance runners, considering the increased number of annual ultra-endurance races and participants in these races [42]. 

Finally, we propose that more research should be conducted on the relationship between oral health and performance in athletes competing in ultra-endurance races, as well as on other factors such as type of diet, specific strength and mountain training programs, and mental strategies that could be adopted by high-performance runners, specifically during longer races.

## 5. Conclusions

Ultra-endurance mountain athletes competing in longer races (>90 km) have more experience and follow harder training schedules than shorter-distance athletes. In longer races, a larger fluid intake before the competition was the single best predictor of performance. For races between 45 and 90 km, training intensity and volume were key predictors of performance, and for races below 45 km, oral health status was a key predictor of performance. Psychological factors, previously reported as good predictors of ultra-endurance mountain race performance, had little influence in the present study.

## Figures and Tables

**Table 1:** Training and psychological variables in G1–G3 athletes.

Variables	Group 1 (M ± SD)	Group 2 (M ± SD)	Group 3 (M ± SD)	F	*p*	Intergroup Comparisons
	*n* = 234	*n* = 79	*n* = 135			
Training-related variables						
Years of mountain race practice	3.0 ± 2.7	3.3 ± 2.7	6.7 ± 4.0	8.963	0.000	1 = 2 < 3
Training per week (h)	6.5 ± 5.0	7.3 ± 3.2	10.7 ± 4.7	3.646	0.030	1 = 2 < 3
Training per session (min)	79.7 ± 42.8	77.4 ± 28.7	102.5 ± 48.7	5.337	0.006	1 = 2 < 3
Training sessions per week	4.1 ± 1.2	4.5 ± 1.2	5.0 ± 1.	7.504	0.001	1 = 2 < 3
Accumulated positive altitude difference (m) per session	542.1 ± 598.9	713.9 ± 908.7	1878.1 ± 1321.1	11.191	0.000	1 < 2 < 3
Accumulated positive altitude difference (m) per week	1347.7 ± 2286.7	1497.7 ± 1845.5	3525.9 ± 2747.8	7.773	0.001	1 = 2 < 3
Marathon best mark (min)	190.7 ± 67.3	220.1 ± 57.9	232.9 ± 70.9	4.830	0.010	1 < 2; 1 < 3
Half-marathon time (min)	98.5 ± 18.3	101.8 ± 38.5	94.2 ± 19.6	0.770	0.466	1 = 2 > 3
Expected time (min)	158.6 ± 91.2	538.0 ± 160.6	1399.4 ± 272.9	426.335	0.000	1 < 2; 1 < 3; 2 < 3
Difference between expected and real time (min)	0.99 ± 33.1	45.0 ± 58.5	112.2 ± 147.9	15.583	0.000	1 < 3; 2 < 3
Psychological variables						
Perceived stress (PPS-14)	17.1 ± 7.0	14.7 ± 6.8	17.3 ± 6.8	0.235	0.791	n.s.
General mental health (AAQ-II score)	14.0 ± 6.4	12.8 ± 6.1	14.0 ± 6.4	0.543	0.583	n.s.

Note: Group 1, race length < 45 km; Group 2, race length between 45 km and 90 km; Group 3, race length > 90 km.

**Table 2:** Predictors of performance for athletes competing in ultra-endurance mountain races, based on race distance.

Variables	Performance Group	Group 1 (M ± SD)	*p*	Group 2 (M ± SD)	*p*	Group 3 (M ± SD)	*p*
Training variables							
Expected race time (min)	Higher	121 ± 37.6		439.8 ± 61.0		1219.3 ± 181.9	
	Lower	194.4 ± 98.1	0.000	590.3 ± 151.1	0.000	1550.3 ± 203.3	0.000
Difference between expected and real time (min)	Higher	−0.39 ± 16.9		33.4 ± 52.2		52.6 ± 103.9	
	lower	4.7 ± 39.1	0.276	56.5 ± 63.1	0.142	169.1 ± 161.8	0.000
Marathon best mark (min)	Higher	183.2 ± 56.6		194.7 ± 55.6		224.1 ± 64.8	
	lower	195.2 ± 76.2	0.491	246.2 ± 59.7	0.008	232.4 ± 64.2	0.604
Half-marathon time (min)	Higher	93.3 ± 10.8		91.2 ± 8.2		90.5 ± 9.8	
	lower	102 ± 18	0.001	106.3 ± 31.3	0.019	94.8 ± 20.2	0.268
Training per week (h)	Higher	7.3 ± 7.6		8.9 ± 3.7		10.6 ± 5.1	
	Lower	6 ± 2.3	0.145	6.2 ± 2.2	0.001	10.4 ± 3.9	0.853
Training per session (min)	Higher	84.5 ± 60.9		80.5 ± 35.4		102.8 ± 50.0	
	Lower	78.2 ± 27.4	0.363	76.6 ± 22.9	0.615	106.1 ± 49.7	0.771
Sessions per week	Higher	4.1 ± 1.1		5.2 ± 1.5		4.8 ± 1.5	
	Lower	4.0 ± 1.1	0.601	4.1 ± 0.9	0.001	4.9 ± 1.2	0.658
Average speed training per week	Higher	10.3 ± 2.8		10.4 ± 3.1		10.2 ± 2.7	
	Lower	9.3 ± 2.8	0.028	8.3 ± 2.9	0.011	9.6 ± 2.2	0.269
Positive change of altitude accumulated per training session (m)	Higher	493.7 ± 523.6		822.3 ± 1345.6		1945.5 ± 1524.8	
	Lower	561 ± 588.5	0.500	543.4 ± 525.5	0.364	1793.0 ± 1138.0	0.627
Positive change of altitude accumulated per week (m)	Higher	1701.8 ± 3644.3		1934.1 ± 2843.5		3595.3 ± 3214.8	
	Lower	1259.1 ± 1174.8	0.351	1200.0 ± 1220.6	0.274	3434.6 ± 2233.9	0.805
Stretching per week (min)	Higher	10.5 ± 5.8		12.7 ± 11.0		11.6 ± 11.0	
	Lower	11.4 ± 8.4	0.411	12.0 ± 5.4	0.764	13.7 ± 11.5	0.392
Stretching sessions per week	Higher	4.1 ± 1.6		4.5 ± 1.8		3.6 ± 2.2	
	Lower	3.8 ± 1.7	0.249	3.7 ± 1.8	0.081	3.4 ± 2.0	0.786
Nutrition/hydration-related variables							
Carbohydrate meals during the week of competition	Higher	4.8 ± 2.2		6.2 ± 3.1		6.3 ± 2.9	
	Lower	5 ± 2.2	0.620	5.4 ± 2.6	0.320	6.5 ± 2.8	0.789
Fluid intake before competition day (l)	Higher	2.5 ± 1.5		2.6 ± 1.0		2.8 ± 1.4	
	Lower	2.5 ± 1.3	0.887	2.5 ± 1.4	0.733	2.2 ± 0.9	0.033
Psychological variables							
Perceived stress (PPS)	Higher	16.2 ± 6.7		14.2 ± 5.9		16.7 ± 6.6	
	Lower	17.1 ± 6.9	0.345	14.5 ± 7.0	0.819	17.5 ± 5.6	0.531
General mental health (AAQII)	Higher	13 ± 5.9		13.0 ± 7.0		14.9 ± 7.0	
	Lower	14.9 ± 6.7	0.055	12.5 ± 5.8	0.792	14.0 ± 5.3	0.498
Oral health status							
Grinding or clenching your teeth while performing your usual tasks	Higher	0.06 ± 0.2		0.1 ± 0.3		0.1 ± 0.3	
	Lower	0.2 ± 0.4	0.004	0.06 ± 0.2	0.331	0.1 ± 0.3	0.967
Nibbling objects	Higher	0.09 ± 0.2		0.1 ± 0.3		0.2 ± 0.4	
	Lower	0.2 ± 0.4	0.036	0.06 ± 0.2	0.184	0.3 ± 0.4	0.235
Biting nails	Higher	0.1 ± 0.3		0.3 ± 0.4		0.3 ± 0.4	
	Lower	0.3 ± 0.4	0.016	0.3 ± 0.4	0.792	0.4 ± 0.5	0.214
Vitamin C pills	Higher	0.01 ± 0.1		0 ± 0		0.1 ± 0.3	
	Lower	0.08 ± 0.2	0.031	0.03 ± 0.1	0.338	0.1 ± 0.3	0.967
Wet pillow upon awakening	Higher	0.09 ± 0.2		0.1 ± 0.3		0.2 ± 0.4	
	Lower	0.2 ± 0.4	0.027	0.09 ± 0.3	0.933	0.1 ± 0.3	0.529
Repeated burps	Higher	0.08 ± 0.2		0.03 ± 0.1		0.1 ± 0.3	
	Lower	0.1 ± 0.3	0.584	0.06 ± 0.2	0.620	0.02 ± 0.1	0.045

Note: Group 1, race length < 45 km; Group 2, race length between 45 km and 90 km; Group 3, race length > 90 km.

## Data Availability

All study data are included in the present manuscript.
